# Baicalin Modulates Hepatic Lipid Metabolism and Improves Intestinal Health in White-Feathered Broilers

**DOI:** 10.3390/biology15181569

**Published:** 2026-09-08

**Authors:** Junxin Li, Xiaowei Huang, Yana Li, Yu Zheng, Shizhong Zhang, Bohan Zheng, Qinjin Li, Xiaohong Huang, Zhaoyan Lin

**Affiliations:** 1 College of Animal Science, Fujian Agriculture and Forestry University, Fuzhou 350002, China; junxin524@fafu.edu.cn (J.L.); hxw18950183114@163.com (X.H.); 52506023059@fafu.edu.cn (Y.L.); zy931891704@163.com (Y.Z.); zhengbohan@fafu.edu.cn (B.Z.); liqinjin0809@163.com (Q.L.); huangxiaohong@fafu.edu.cn (X.H.); 2 University Key Laboratory for Integrated Chinese Traditional and Western Veterinary Medicine and Animal Healthcare in Fujian Province, Fujian Key Laboratory of Traditional Chinese Veterinary Medicine and Animal Health, Fujian Agriculture and Forestry University, Fuzhou 350002, China; 3 Institute of Animal Husbandry and Veterinary Medicine, Fujian Academy of Agricultural Sciences, Fuzhou 350013, China; zhangshizhong@faas.cn

**Keywords:** baicalin, white-feathered broilers, antioxidation, lipid metabolism, gut microbiota, plasma metabolomics

## Abstract

Liver and intestinal health govern the production performance of broilers. Baicalin (BA) is a flavonoid extracted from the dried roots of *Scutellaria baicalensis* and possesses a wide range of biological activity. In this study, 66 one-day-old white-feathered broilers were selected and randomly divided into two groups. Broilers in the control group were fed a basal diet, whereas the BA group received the basal diet supplemented with 100 mg/kg BA. The results showed that BA supplementation markedly elevated the antioxidant capacity of the serum and liver (*p* < 0.05). Meanwhile, the villus height/crypt depth ratio and the activity of intestinal digestive enzymes were significantly higher in the BA group (*p* < 0.01). Transcriptomics, RT-qPCR, and Western blot assays revealed that BA-mediated effects were associated with the hepatic PPARα signaling pathway and the regulation of lipid metabolism. Furthermore, a combined analysis encompassing intestinal microbiota 16S rRNA sequencing and plasma metabolomics revealed that the BA-induced enrichment of multiple bacterial taxa, such as *Lachnospiraceae*, may modulate butyrate metabolism and circulating metabolites including isoleucyl-glutamate in broilers. In conclusion, dietary BA supplementation improved the hepatic and intestinal health of white-feathered broilers, reshaped the intestinal microbiota composition, and altered the plasma metabolite profile, with these benefits linked to the PPARα signaling pathway.

## Simple Summary

Fast-growing meat chickens often suffer from liver and digestive tract damage caused by stress, which hinders their health and growth. Baicalin, an active constituent of *Scutellaria baicalensis*, exhibits diverse biological activity. In this study, dietary supplementation with 100 mg/kg baicalin in white-feathered broilers enhanced the hepatic antioxidant capacity and modulated hepatic lipid metabolism via the PPARα signaling pathway. Meanwhile, baicalin increased intestinal digestive enzyme activity, reshaped the intestinal microbiota composition, and altered plasma metabolite profiles. These findings provide a reference for the clinical application of baicalin to improve hepatic and intestinal health in broilers.

## 1. Introduction

The white-feathered broiler industry holds a crucial position in the global meat production and supply system. With improvements in the scale of intensive farming, the growth performance, quality, and safety of broilers have been significantly enhanced. In the face of various environmental pressures, the breeding and production of white-feathered broiler chickens often also encounter various challenges [1]. Improper feeding management and other environmental factors can induce various stress responses and injuries in broiler chickens [2]. As core metabolic organs in poultry, the liver and intestine are highly sensitive to stress stimuli; impairments in their physiological functioning directly impede broiler growth and undermine the health of the organism [2]. To date, several strategies have been developed to mitigate such stress, including genetic improvement [3], environmental control [4], and dietary additives [5]. However, these issues remain unresolved and have led to an increase in the mortality rates of broiler chickens, seriously affecting breeding efficiency [6].

Due to their rapid growth rates, broiler chickens are more susceptible to external stimuli [7], which induces oxidative stress that impairs mitochondria, cell membranes, and proteins. The liver serves as the primary antioxidant organ in broilers, functioning to scavenge reactive oxygen species, produce antioxidants, and repair oxidative damage. It synthesizes key antioxidant enzymes including superoxide dismutase (SOD), glutathione peroxidase (GSH-Px), and catalase (CAT), as well as the non-enzymatic antioxidant glutathione (GSH), to mitigate oxidative stress-induced cell apoptosis and tissue injury [8]. Meanwhile, the liver is also the main organ for lipid metabolism in broilers [9]. Oxidative stress can trigger hepatic lipid-metabolic disorder, suppress lipid catabolism, induce lipid accumulation, and consequently injure the liver, thereby impairing broiler health [10].

The intestine is the main site for nutrient digestion and absorption in broiler chickens. The activity of digestive enzymes in the intestine is a key indicator for evaluating the integrity of intestinal development, digestive function, and the health status of the body [11]. Among them, α-glucosidase is located on the brush border of the small-intestinal mucosa and can further break down the oligosaccharides produced by the hydrolysis of starch into glucose, determining the final absorption efficiency of carbohydrates. Lipase is mainly responsible for the hydrolysis of dietary triglycerides, releasing fatty acids and monoglycerides for the body to absorb and utilize; trypsin is secreted by the pancreas and acts on the intestine, which can decompose large-molecule proteins in feed, generating absorbable small peptides and free amino acids [11,12]. Decreased digestive enzyme activity impairs nutrient metabolism. Undigested nutrients accumulated in the intestinal lumen facilitate the overgrowth of harmful bacteria, thereby triggering intestinal mucosal inflammation and eventually retarding broiler growth [13].

The gut–liver axis represents a source of bidirectional crosstalk between the intestine and liver, mainly mediated by portal vein-derived microbial metabolites, bile acid enterohepatic circulation, and immune mediators. Intestinal metabolites, lipopolysaccharides, and other microbial products are transported to the liver via portal blood, regulating the hepatic antioxidant capacity, lipid metabolism, and inflammatory responses; in turn, the liver secretes bile acids and antimicrobial substances into the intestinal tract to modulate the microbiota composition [14]. Under oxidative stress conditions, an impaired intestinal barrier and gut dysbiosis disrupt gut–liver homeostasis, triggering hepatic lipid disorder and oxidative injury in broilers. Targeting the gut–liver crosstalk via nutritional intervention provides a feasible strategy to improve the hepatic and intestinal health of broilers.

Baicalin (BA) is a flavonoid substance extracted from the dried roots of *Scutellaria baicalensis* [15]. In vitro and in vivo studies have confirmed that BA possesses multiple biological actions, including antioxidative [16,17,18], anti-inflammatory [19], anti-tumor [20,21], and antiviral [22] properties. In the poultry industry, BA relieved H_2_O_2_-triggered oxidative damage in DF-1 cells and preserved mitochondrial function via the Mfn2-mediated mitochondria-associated membrane (MAM)/Ca^2+^ signaling axis [17]. Dietary BA supplementation alleviated aflatoxin B1-triggered growth retardation and hepatic injury in ducklings, as evidenced by restored serum hepatic biomarkers and improved hepatic histomorphology [23]. However, reports on systematic studies exploring the synergistic regulatory effects of BA on antioxidant homeostasis, hepatic lipid metabolism, and intestinal digestive function in white-feathered broilers are still relatively limited.

This study aimed to evaluate the influences of BA on growth performance and hepatic and intestinal health in broilers, as well as its underlying mechanisms. The results provide a reference for the application of BA as a feed additive in the healthy breeding of broiler chickens.

## 2. Materials and Methods

### 2.1. Animal Treatments

The animal experiment was reviewed and approved by the Animal Ethics Committee of Fujian Agriculture and Forestry University (approval code: PZCASFAFU24029), according to the guidelines for Laboratory Animal Use and Care from the Chinese Center for Disease Control and Prevention and the Rules for Medical Laboratory Animals in 1998 from the Chinese Ministry of Health.

A total of 66 one-day-old white-feathered broiler chickens (strain: Shengze 901) were purchased from Shengze Co., Ltd. (Zixi, China) and randomly divided into two groups; each group contained 33 birds. All broilers were housed in wire-mesh cages (1.2 × 0.8 × 0.5 m) under controlled environmental conditions. The room temperature was maintained at 33 °C for the first 3 days and then gradually decreased by 2–3 °C per week until it reached 24 °C, which was maintained for the remainder of the trial. The relative air humidity was kept at 55–65%. A 23 h light/1 h dark lighting program was applied during the whole experimental period. All chickens received routine commercial vaccinations according to standard broiler immunization protocols. The control group was supplied with an unsupplemented basal diet. For the experimental treatment group, BA was incorporated into the basal diet as an additional supplement at a concentration of 100 mg/kg. All broilers had ad libitum access to feed and clean water for the entire 42-day experimental feeding period. BA was purchased from MedChemExpress Inc. (Monmouth Junction, NJ, USA, purity > 95%, molecular formula: C_21_H_18_O_11_); the chemical structure of BA is presented in Supplementary Figure S1. The basic feed was formulated in accordance with the national standard of the People’s Republic of China (GB/T 5916–2020 [24]). The composition and nutritional levels of the basal diet are detailed in Table 1.

On days 21 and 42 of the trial, the body weights of broilers in each group were recorded to calculate the average daily gain (ADG) and average daily feed intake (ADFI). The feed conversion ratio (FCR) was further computed using the formula FCR = ADFI/ADG.

Blood, liver, and intestinal tissues were harvested on days 21 and 42. Six broilers from each group were randomly selected and euthanized for sample collection on day 21, and all remaining broilers were slaughtered on day 42. Broilers were anesthetized via the intramuscular injection of thiopental sodium (0.7 mL/kg) prior to blood collection. After blood collection, liver and intestinal samples were collected. Portions of these tissues were fixed in 4% paraformaldehyde solution for morphological analysis. The remaining tissue aliquots were snap-frozen in liquid nitrogen and stored at −80 °C for subsequent analyses.

### 2.2. Antioxidant Assays

Liver tissues were homogenized with pre-cooled extraction solution under an ice bath and centrifuged at 12,000× *g* for 5 min at 4 °C, and the supernatant was collected and placed on ice for measurement. The liver supernatant and serum were separately used to determine the total antioxidant capacity (TAC, KTB1500), malondialdehyde (MDA, KTB1050) content, superoxide dismutase (SOD, KTB1030) activity (the reagent kits were purchased from Abbkine Scientific Co., Ltd., Wuhan, China), and glutathione peroxidase (GSH-Px, G0204W, Greist Biotechnology Co., Ltd., Suzhou, China). All assays were conducted in accordance with the manufacturer’s protocols. There were n = 4 biological replicates per treatment; each biological sample was analyzed in 3 technical replicates.

### 2.3. Morphological Assay

The liver and intestinal specimens were fixed at room temperature with 4% paraformaldehyde for 48 h and processed for routine paraffin embedding and serial sectioning. Sections of 4 μm thickness were stained with hematoxylin–eosin (H&E). Images were captured with a brightfield digital microscope. There were n = 3 biological replicates per treatment.

### 2.4. Intestinal Digestive Enzyme Detection

The contents of the jejunum were homogenized on ice and centrifuged at 15,000× *g* for 10 min at 4 °C; the supernatant was used to detect the activity of α-glucosidase (KTB1015), trypsin (KTB2320), and lipase (KTB2241), in accordance with the manufacturer’s protocols. All reagents were purchased from Abbkine Scientific Co., Ltd. (Wuhan, China). There were n = 4 biological replicates per treatment; each biological sample was analyzed in 3 technical replicates.

### 2.5. Transcriptome Sequencing

Transcriptome sequencing was conducted by Novogene Co., Ltd. (Beijing, China). Total RNA purification and reverse transcription were performed following the protocols of the corresponding reagent kits. RNA library construction and high-throughput sequencing were conducted on the Illumina platform (Illumina, San Diego, CA, USA), in strict accordance with the manufacturer’s instructions. To identify differentially expressed genes (DEGs) between two different samples, the expression levels of the different samples were calculated using the transcripts per million reads (TPM) method. The sequencing work was completed on the NovaSeq 6000 platform (Illumina, San Diego, CA, USA), with n = 4 biological replicates per treatment. Differential expression analysis was performed using DESeq2 (v1.34.0). The Benjamini–Hochberg algorithm was used for multiple-testing correction to obtain FDR-adjusted q-values. Genes with FDR < 0.05 and |log_2_FoldChange| ≥ 1.0 were regarded as significantly differentially expressed genes for subsequent GO and KEGG enrichment. The volcano plot was drawn based on unadjusted raw *p*-values (raw *p* ≤0.05, |log_2_FoldChange| ≥1.0) for intuitive visualization. The transcriptome sequencing data reported in this article have been submitted to the NCBI Sequence Read Archive (SRA) with the accession number PRJNA1506322.

### 2.6. 16S rRNA Sequencing for Microbiota Analysis

Total microbial genomic DNA was extracted from broiler cecal contents. The bacterial 16S rRNA V3–V4 region was amplified by PCR, and library sequencing was conducted on the NovaSeq 6000 platform by Novogene Co., Ltd. (Beijing, China). Raw reads were quality-controlled, spliced, and de-chimerized to acquire effective tags, which were clustered into OTUs at 97% similarity using UPARSE (v7.0.1001). Taxonomic annotation was performed based on the SILVA 138.2 database. The QIIME software (v1.9.1) was used for alpha diversity, beta diversity, and species composition analyses; LEfSe (v1.1.2) was used to screen differential bacterial taxa between groups. There were n = 4 biological replicates per treatment. The transcriptome sequencing data reported in this article have been submitted to the NCBI Sequence Read Archive (SRA) with the accession number PRJNA1506411.

### 2.7. Untargeted Metabolomics Analysis

Untargeted metabolomics analysis was performed on plasma metabolites extracted with 80% methanol solution. UPLC-MS/MS detection was conducted by Novogene Co., Ltd. (Beijing, China) under dual positive/negative ion modes with QC and blank samples for quality control. Raw mass spectrum data were processed with XCMS for peak extraction, alignment, and normalization; metabolites with CV > 30% in QC samples were excluded. Metabolites were annotated against the NovoMetDB, HMDB, and KEGG databases. Differential metabolites were screened by combining the VIP value, *p*-value, and fold change, followed by KEGG functional enrichment to compare plasma metabolic profiles among groups. There were n = 3 biological replicates per treatment.

### 2.8. Real-Time Quantitative PCR (RT-qPCR)

RNA extraction from liver tissues was performed using the AG RNAex Pro Kit (AG21101, Accurate Biotech, Changsha, China). The 260/280 nm absorbance ratio of the samples was measured to determine the total RNA concentration. RNA reverse transcription was carried out using the ABScript Neo RT Master Mix for qPCR with the gDNA Remover Kit (RK20433, Wuhan AoboTech Biotechnology Co., Ltd., Wuhan, China), according to the instructions. The mRNA expression levels of the target genes were detected using a real-time fluorescence quantitative PCR instrument (CFX96, Bole Life Medical Products Co., Ltd., Shanghai, China). The RT-qPCR reaction program was set as follows: 3 min of pre-denaturation at 95 °C, followed by 40 amplification cycles. Each cycle consisted of 5 s of denaturation at 95 °C, 30 s of annealing, and extension at 60 °C, and the fluorescence signal was collected at the end of each extension. The relative expression levels of each gene mRNA were calculated using the 2^−ΔΔCt^ method, and the primer sequences are shown in Supplementary Table S1. There were n = 4 biological replicates per treatment; each biological sample was analyzed in 3 technical replicates.

### 2.9. Western Blot Analysis

Proteins were extracted from liver tissues using RIPA lysis buffer containing protease inhibitors. Protein quantification was performed using the BCA assay (Aixin Biotechnology Co., Ltd., Wuhan, China), and the same amount of protein was electrophoresed in SDS polyacrylamide gel and transferred onto a PVDF membrane (Cytiva Life Sciences, Marlborough, MA, USA). The primary antibody was incubated overnight at 4 °C: anti-FASN (1:1000, NB400-114, Novus Biologicals, Centennial, CO, USA), anti-PPARα (1:2000, YM8234, Immunoway, San Jose, CA, USA), anti-p-PPARα (1:1000, YP1576, Immunoway, San Jose, CA, USA), anti-HSL (1:2000, A94794, Nature Biosciences, Hangzhou, China). Then, the secondary antibody (RGARO01, Proteintech, Rosemont, IL, USA, 1:10,000) was incubated at room temperature for 1 h. The samples were exposed under a chemiluminescence imaging analysis system (ChemiScope 6100, CLINX, Shanghai, China), and data analysis was completed using ImageJ (Version 1.54f, National Institutes of Health, Bethesda, MD, USA). There were n = 3 biological replicates per treatment.

### 2.10. Gas Chromatography–Mass Spectrometry

The butyric acid concentrations in the plasma and cecal contents were quantified using a Shimadzu GC-MS 2010 instrument (Shimadzu Corporation, Kyoto, Japan) equipped with a KB-FFAP capillary column (60 m × 0.25 mm × 0.32 μm). A 1.0 mg/mL stock standard solution was prepared by dissolving 1.0 mg accurately weighed butyric acid standard (purity > 98.0%, Yuanye Bio-Technology Co., Ltd., Shanghai, China) in methanol (chromatographic grade) to a constant volume of 1 mL, which was sealed and stored at 0–4 °C, and serial working standard solutions were freshly diluted with methanol before use. For sample pretreatment, 0.2 g cecal content or 0.2 mL plasma was transferred into a centrifuge tube, mixed with 200 μL 50% sulfuric acid (analytical grade) for acidification, supplemented with diethyl (chromatographic grade) ether to a total volume of 2 mL, vortexed thoroughly for 2 min, and centrifuged at 12,000 rpm and 4 °C for 10 min, and the supernatant ether layer was filtered prior to injection. The GC program used helium as a carrier gas, with an initial column temperature of 40 °C held for 2 min, followed by heating to 150 °C at 15 °C/min with a 1 min hold; this was followed by ramping up to 250 °C at 30 °C/min and maintaining for 5 min. The injector temperature was set at 250 °C, the injection volume was 1 μL, the split ratio was 1:1, and the MS parameters included an ion source temperature of 230 °C, interface temperature of 250 °C, and solvent delay of 3 min. Chromatographic data collection and peak integration were performed via the Shimadzu GCMSsolution software (GC-MS 2010, Shimadzu Corporation, Kyoto, Japan), and external standard curves were applied to calculate the butyric acid levels in the plasma (μg/mL) and cecal contents (μg/g wet weight). There were n = 3 biological replicates per treatment.

### 2.11. Statistical Analysis

Data were expressed as the mean ± standard deviation (SD). Statistical analysis was performed using the GraphPad Prism 10 software (version 10, GraphPad Software Inc., San Diego, CA, USA) and SPSS 26.0 software (version 26.0, IBM Inc., New York, NY, USA). A two-tailed unpaired Student’s *t*-test was used when variances were homogeneous according to the F-test. The two-tailed unpaired *t*-test with Welch’s correction was applied in cases of unequal variance. A *p* value of 0.05 or less indicated statistical significance.

## 3. Results

### 3.1. Growth Performance of Broilers Fed Diets Supplemented with BA

As shown in Table 2, dietary supplementation of 100 mg/kg BA altered the 21-day final body weight (FBW) of broilers from 856.52 ± 2.68 g (control) to 886.8 ± 41.4 g and the 42-day FBW from 3070.31 ± 32.41 g (control) to 3044.26 ± 82.87 g. Meanwhile, BA supplementation decreased the final feed conversion ratio (FCR) from 1.38 ± 0.01 to 1.33 ± 0.03. However, none of these differences reached statistical significance (*p* > 0.05).

### 3.2. BA Improves Serum and Hepatic Antioxidant Capacity in Broilers

**Figure 1:**
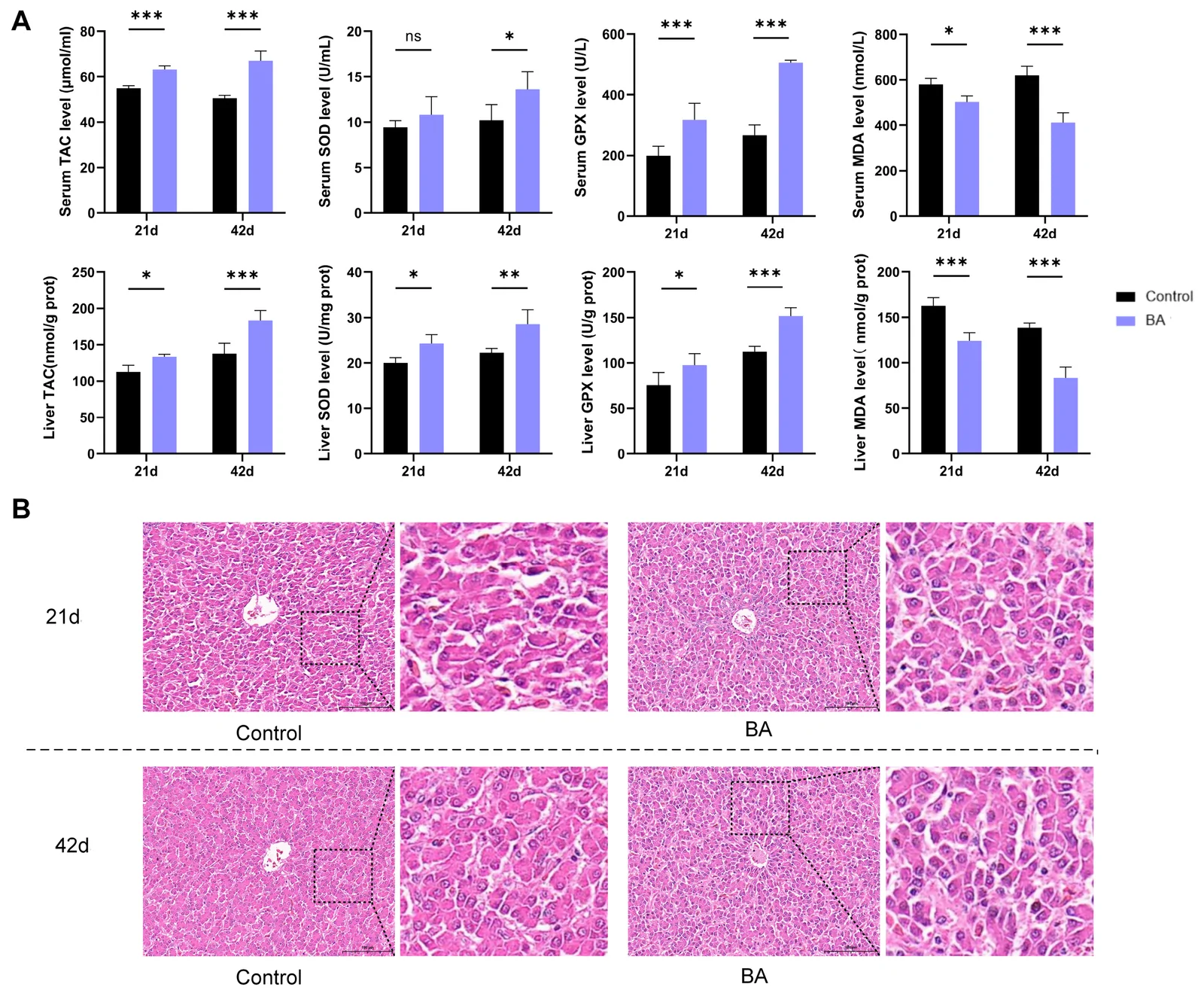
BA improves serum and hepatic antioxidant capacity in broilers. (**A**) The total antioxidant capacity (TAC), superoxide dismutase (SOD), glutathione peroxidase (GPX), and malondialdehyde (MDA) quantitative detection results in the serum and liver at 21 and 42 days of age. n = 4 biological replicates per treatment. (**B**) The H&E staining of liver tissues. Liver sections were observed under an optical microscope at 200× magnification. Scale bar = 100 μm. The dotted box indicates the magnified region of the hepatic parenchyma. n = 3 biological replicates per treatment. * *p* < 0.05, ** *p* < 0.01, *** *p* < 0.001, ns, non-significant. BA, baicalin.

To evaluate the antioxidant capacity of BA, the total antioxidant capacity (TAC), superoxide dismutase (SOD), glutathione peroxidase (GPX), and malondialdehyde (MDA) in the serum and liver were measured. As shown in Figure 1A, compared with the control group, broilers in the BA group exhibited significantly higher serum and hepatic TAC as well as GPX activity at day 21 (mid-feeding stage) and day 42 (finishing stage, *p* < 0.05). At day 21, serum SOD activity was numerically elevated in the BA group relative to the controls, without statistical significance (*p* > 0.05). By contrast, hepatic SOD at day 21, together with both serum and hepatic SOD at day 42, was significantly increased in the BA group (*p* < 0.05). Moreover, the serum and hepatic MDA content was remarkably lower in BA-treated broilers at both 21 and 42 days (*p* < 0.05).

The histopathological observation of liver tissues revealed a similar hepatic morphology between the two groups on day 21 and day 42, with neatly arranged hepatic cords and no obvious pathological lesions in hepatocytes (Figure 1B). Collectively, these data demonstrate that BA effectively enhances the serum and hepatic antioxidant status in broilers and causes no apparent histological damage to the liver.

### 3.3. BA Modulates Intestinal Histological Traits and Digestive Enzyme Activity in Broilers

**Figure 2:**
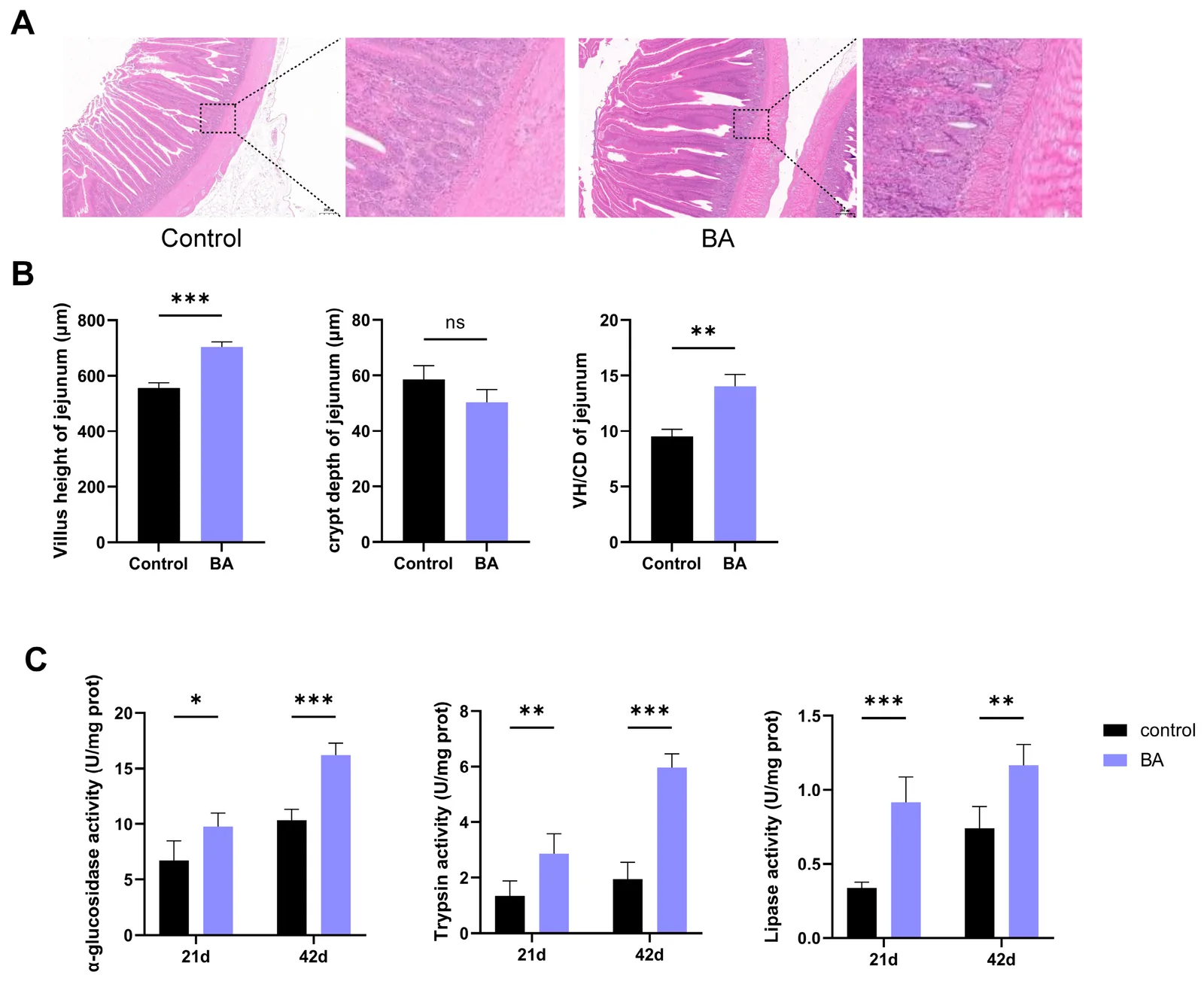
BA enhances intestinal absorption in broilers. (**A**) H&E staining of the jejunum tissue in broilers at 42 days. Jejunum sections were visualized using an optical microscope at 100× magnification. Scale bar = 250 μm. The dotted box denotes the region selected for subsequent high-magnification observation. n = 3 biological replicates per treatment. (**B**) Quantitative statistical results for villus height, crypt depth, and the ratio of villus height to crypt depth (VH/CD). n = 3 biological replicates per treatment. (**C**) Detection results for intestinal α-glucosidase, trypsin, and lipase activity in broilers at 21 and 42 days. n = 4 biological replicates per treatment. * *p* < 0.05, ** *p* < 0.01, *** *p* < 0.001, ns, non-significant. BA, baicalin.

The results of the intestinal H&E staining are presented in Figure 2A. At 42 days of age, the jejunal villi were longer, plumper, and more regularly arranged in the BA group, with an intact villous epithelium free of severe exfoliation and well-organized crypt structures, as compared with the control group. As shown in Figure 2B, the villus height was significantly elevated in BA-supplemented broilers relative to the controls (*p* < 0.001); the crypt depth remained unaffected by BA treatment (*p* > 0.05). The villus height/crypt depth ratio (VH/CD) was significantly greater in the BA group (*p* < 0.01).

The digestive enzyme activity in the jejuna of broilers was also detected. As shown in Figure 2C, BA significantly elevated the activity of α-glucosidase, trypsin, and lipase at both 21 and 42 days (*p* < 0.05). These results reveal that BA boosts intestinal digestive enzyme activity and improves the intestinal nutrient absorption capacity, thereby enhancing the overall digestive and absorptive functioning of the intestine.

### 3.4. BA Modulates Hepatic Antioxidant and Lipid Metabolic Signaling in White-Feathered Broilers

**Figure 3:**
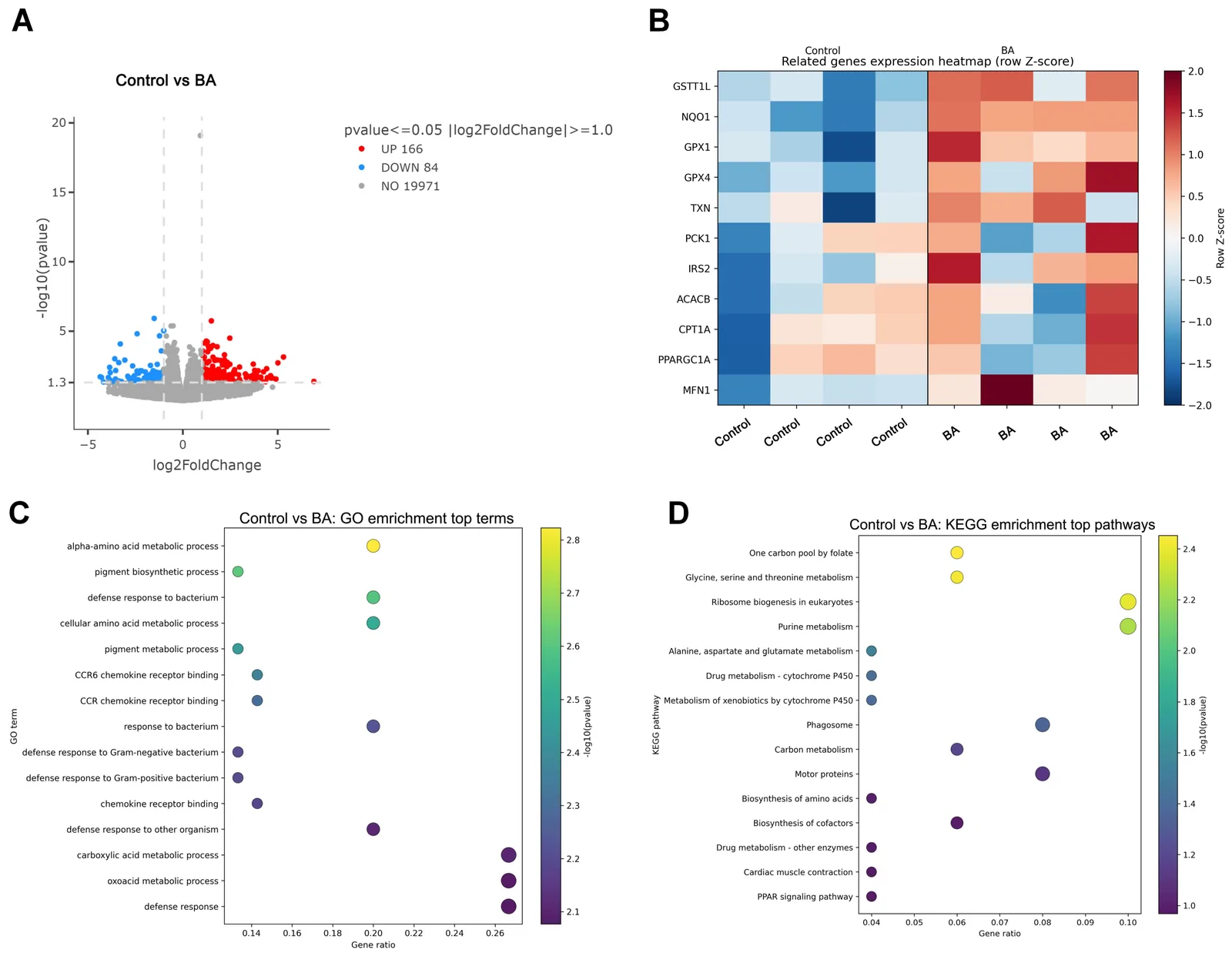
BA modulates hepatic antioxidant and lipid metabolic signaling in white-feathered broilers. (**A**) Volcano plot of differentially expressed genes (DEGs). The x-axis indicates the log_2_FoldChange, and the y-axis represents the −log_10_ (raw *p*-value). Red dots denote upregulated genes, and blue dots represent downregulated genes, plotted with the threshold of raw *p* ≤ 0.05 and |log_2_FoldChange| ≥ 1.0. Grey dots indicate genes with no significant expression alteration. n = 4 biological replicates per treatment. (**B**) Row Z-score-normalized heatmap of antioxidant and lipid metabolism-related differentially expressed genes. n = 4 biological replicates per treatment. (**C**) Bubble chart showing enriched GO terms for differentially expressed genes. n = 4 biological replicates per treatment. (**D**) Bubble chart showing enriched KEGG pathways for differentially expressed genes. n = 4 biological replicates per treatment. BA, baicalin.

To explore the antioxidant mechanism of BA, transcriptome sequencing was performed on liver tissues, and over 20,000 genes were screened. Compared with the control group, 166 genes were upregulated and 84 genes were downregulated in the livers of broilers in the BA group (Figure 3A). Differentially expressed genes included antioxidant-related genes such as *GSTT1L*, *NQO1*, and *GPX*, as well as lipid metabolism-regulating genes including *ACC*, *CPT1A*, and *PPAR* (Figure 3B). GO enrichment indicated the significant enrichment of carboxylic acid and amino acid metabolic processes (Figure 3C), and KEGG analysis identified the PPAR signaling pathway and cytochrome P450-related xenobiotic metabolism pathways (Figure 3D). These enriched functional terms and pathways were consistent with the expression changes of the differentially expressed genes.

**Figure 4:**
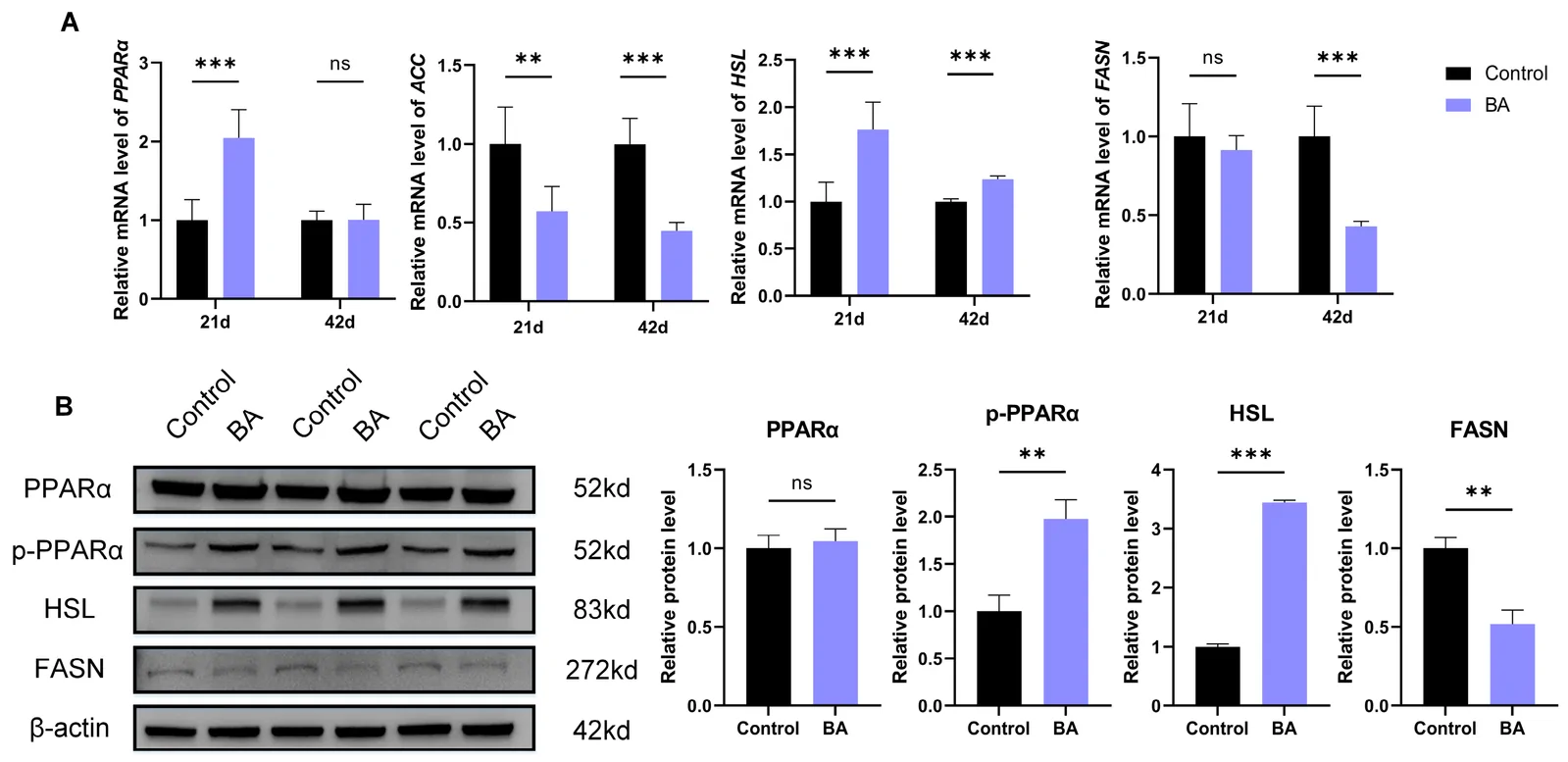
BA regulates the PPARα-related signaling pathway in broilers. (**A**) The relative mRNA expression levels of *PPARα*, *ACC*, *HSL*, and *FASN* in liver samples collected at 21 and 42 days. n = 4 biological replicates per treatment. (**B**) The protein expression levels of phosphorylated PPARα, ACC, HSL, and FASN in the liver at 42 days. n = 3 biological replicates per treatment. ** *p* < 0.01, *** *p* < 0.001, ns, non-significant. BA, baicalin.

Furthermore, the transcriptomic results were validated by RT-qPCR (Figure 4A) and Western blot assays (Figure 4B). The results demonstrated that the hepatic expression of *ACC* was significantly lower (*p* < 0.01), while *HSL* expression was markedly higher (*p* < 0.001), in the BA group compared with the control group at both 21 d and 42 d. The mRNA level of *PPARα* was significantly upregulated in the BA group at 21 d (*p* < 0.001), whereas no significant difference was observed at 42 d (*p* > 0.05). The expression of *FASN* tended to decrease without statistical significance at 21 d (*p* > 0.05) and was significantly reduced at 42 d (*p* < 0.001). The Western blot results revealed that the ratios of FASN were significantly decreased (*p* < 0.01), whereas phosphorylated PPARα and the HSL protein level were significantly elevated (*p* < 0.001) in the BA group at 42 d. Collectively, these findings indicate that BA regulates hepatic lipid metabolism via modulating the PPARα-related signaling pathway in broilers.

### 3.5. BA Improves Intestinal Microbiota Homeostasis and Modulates Plasma Metabolites

**Figure 5:**
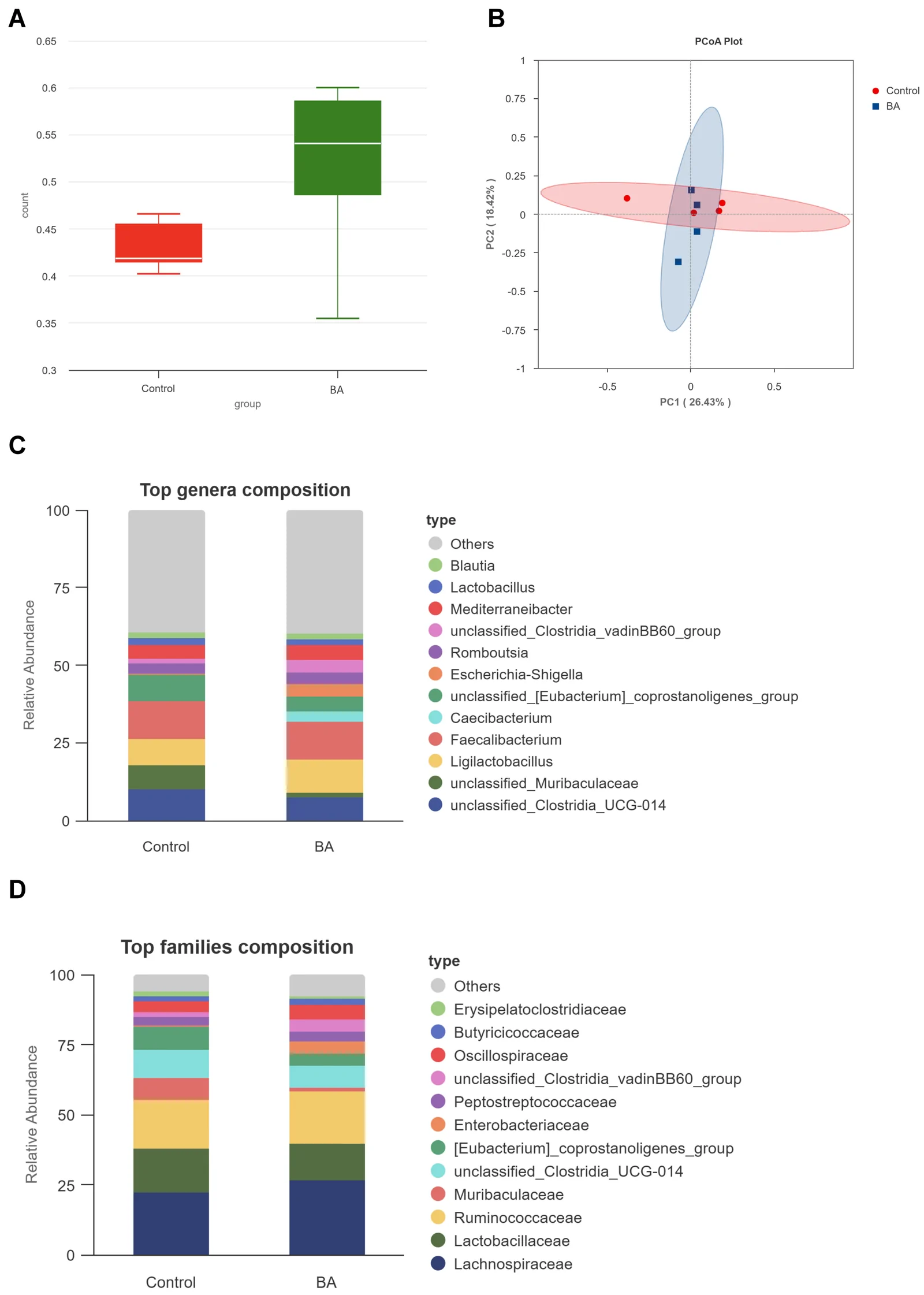
BA effectively modulated the composition of the cecal microbiota in broilers. (**A**) The α-diversity index (count) between the control and BA groups. n = 4 biological replicates per treatment. (**B**) The principal coordinate analysis (PCoA) plot based on the Bray–Curtis distance, showing the β-diversity separation of the gut-bacterial communities between the two groups. PC1 and PC2 explained 26.43% and 18.42% of the total variance, respectively. n = 4 biological replicates per treatment. (**C**) The relative abundances of the bacterial genera in the intestinal microbiota. n = 4 biological replicates per treatment. (**D**) The relative abundances of the bacterial families at the family taxonomic level. n = 4 biological replicates per treatment. PC, principal component; PCoA, principal coordinate analysis; BA, baicalin.

To evaluate the regulatory effects of BA on the intestinal microbiota and metabolites of broilers, 16S rRNA and non-targeted metabolomics analyses were conducted, followed by their integrated analysis. The alpha diversity analysis showed that the species richness index exhibited an increasing trend in the BA group compared with the control group, although the difference was not statistically significant (*p* > 0.05, Figure 5A). For β-diversity, principal coordinate analysis (PCoA) based on the Bray–Curtis dissimilarity exhibited a trend of separation among the gut-bacterial communities from the two groups, with PC1 and PC2 contributing 26.43% and 18.42% of the total variance, respectively (Figure 5B). From the perspective of the 16S bacterial community composition, there were differences in multiple high-abundance bacterial groups between the control and BA groups (Figure 5C,D). At the genus level, the BA group showed a markedly increased relative abundance of *Caecibacterium* in the cecal contents. Meanwhile, the abundances of *Ligilactobacillus*, *Lactobacillus*, and *Blautia* showed an upward trend in the BA group; these have been reported to produce short-chain fatty acids and exert beneficial effects on intestinal health. In contrast, the relative proportions of *Mediterranebacter* and *Romboutsia* were decreased in the BA group compared with the control group. At the family level, the BA intervention elevated the relative abundances of *Lachnospiraceae* and *Butyricicoccaceae*, whereas the abundance of *Muribaculaceae* was reduced. These findings indicate that BA effectively modulated the composition of the cecal microbiota at both the family and genus taxonomic levels in broilers.

**Figure 6:**
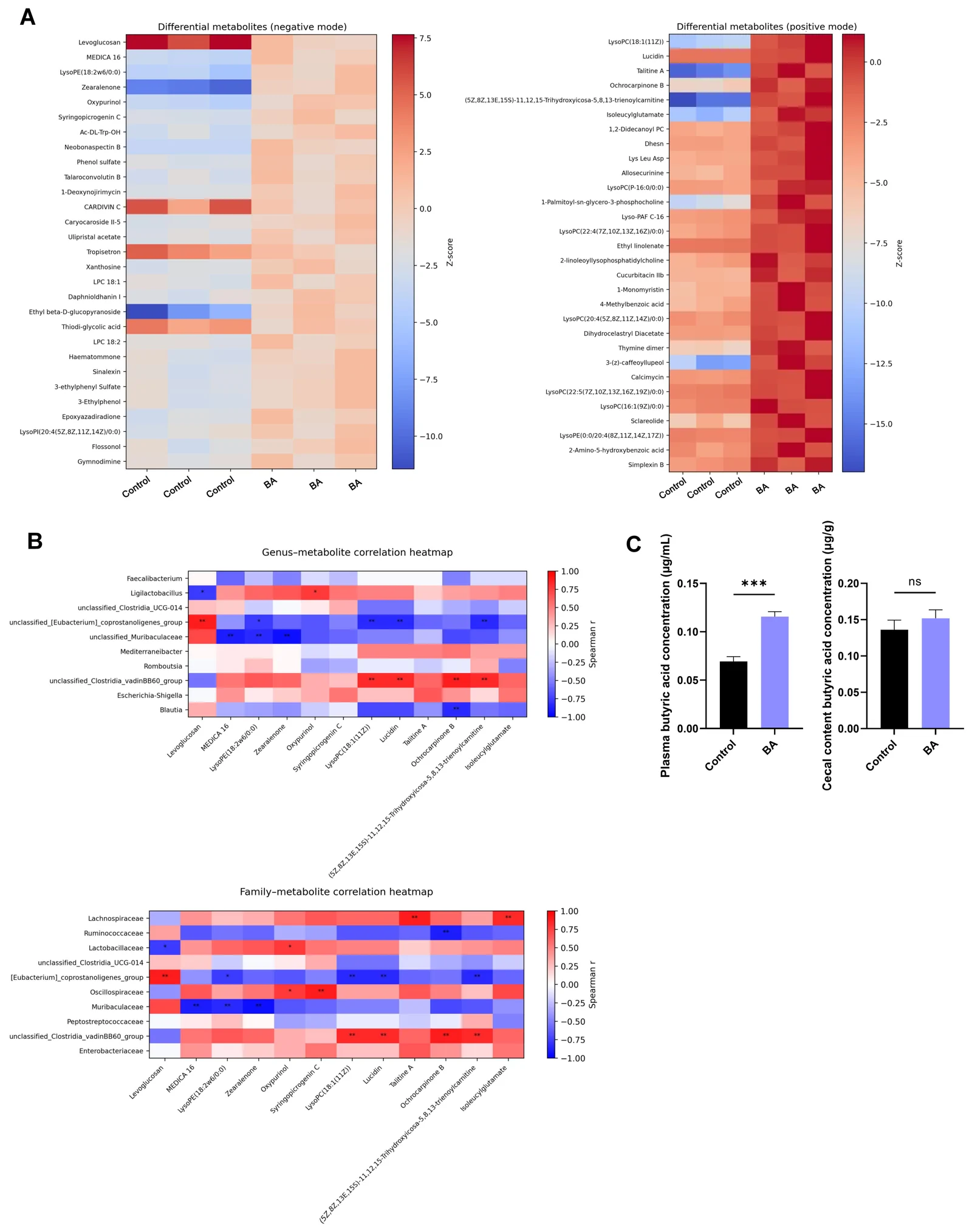
Non-targeted metabolomics analyses and microbiota–metabolite correlation analysis. (**A**) Row Z-score heatmap of differential metabolites identified under negative and positive ion modes. n = 3 biological replicates per treatment. (**B**) Spearman correlation heatmap of dominant intestinal bacterial genera and families versus differential metabolites. Red indicates positive correlation, and blue indicates negative correlation. n = 3 biological replicates per treatment. Note that these correlational findings do not represent causal effects. (**C**) Butyric acid concentrations in plasma and cecal contents at 42 days. n = 3 biological replicates per treatment. * *p* < 0.05, ** *p* < 0.01, *** *p* < 0.001, ns, non-significant. LysoPE, lyso-phosphatidylethanolamine; LPC, lysophosphatidylcholine; LysoPI, lyso-phosphatidylinositol; PC, phosphatidylcholine; Lyso-PAF, lyso-platelet-activating factor; Lys, lysine; Leu, leucine; Asp, aspartic acid; BA, baicalin.

From a metabolomic perspective, various lipid species and redox-related metabolites were screened as differential metabolites. Levoglucosan, zearalenone, xanthosine, and LPC species constituted representative differential metabolites in negative ionization mode, whereas *lysophosphatidylcholines* and *isoleucylglutamate* dominated the differentially expressed metabolites in positive ionization mode (Figure 6A).

Spearman’s correlation analysis was performed to uncover the associations between gut-bacterial families and differential plasma metabolites. The results revealed that *Lachnospiraceae* exhibited a significant positive correlation with isoleucyl-glutamate (*p* < 0.05). The BA-induced enrichment of *Lachnospiraceae* is potentially associated with host amino acid metabolism and circulating isoleucyl-glutamate levels in broilers. Meanwhile, LysoPE (18:2w6/0:0) exhibited a significant negative correlation with *Muribaculaceae* (*p* < 0.05), LysoPC (18:1(11Z)) was negatively associated with the *coprostanoligenes* group, and the acylcarnitine metabolite (5Z,8Z,13E,15S)-11,12,15–Trihydroxycosa-5,8,13-trienoylcarnitine showed a significant positive correlation with *Lachnospiraceae*, whereas it was negatively correlated with *Ruminococcaceae* (Figure 6B).

Since isoleucyl-glutamate is closely associated with the biosynthesis and metabolism of butyrate, the butyric acid concentrations in the intestinal contents and plasma were further evaluated (Figure 6C). At 42 days of age, broilers in the BA group exhibited significantly higher plasma butyric acid levels than those in the control group (*p* < 0.001). Compared with the control group, the butyric acid concentrations in the cecal contents showed an increasing trend in the BA group, yet no significant difference was observed (*p* > 0.05). Collectively, these results suggest that BA enhances intestinal microbiota homeostasis and remodels plasma metabolic profiles, accompanied by elevated plasma butyric acid concentrations.

## 4. Discussion

BA has been demonstrated to promote the growth of yellow broiler chickens at appropriate supplemental dosages [25]. Although dietary BA supplementation numerically reduced the FCR without statistical significance in the present study, minor improvements in feed conversion efficiency are still of great practical significance for commercial broiler production, as the feed cost accounts for the major proportion of the total broiler-raising expenditure [26]. Even a small reduction in the FCR could effectively decrease the feed cost per kilogram of body weight gain and improve economic returns under large-scale farming conditions. Further commercial-scale feeding trials are required to verify its actual economic benefits. Meanwhile, increased jejunal digestive enzyme activity might have been one of the contributors to the numerical improvement in broiler growth performance in this study.

Previous studies have confirmed that dietary supplementation with 100 mg/kg BA could mitigate the aflatoxin B1-induced reduction in growth performance in ducklings [23]; supplementing 150–450 mg/kg BA in the diet could ameliorate deoxynivalenol-triggered intestinal damage in broiler chickens [27]. In the present study, 100 mg/kg BA was supplemented in the broiler diet. This dosage was comprehensively determined based on previous studies [23,27,28]. Whether other doses of BA could achieve better effects remains to be verified in further experiments.

Oxidative stress represents one of the major challenges in commercial broiler production [29]. The defense of the antioxidant system mainly depends on enzymatic systems to eliminate excessive reactive oxygen species (ROS) and block lipid peroxidation reactions [25,30]. In the present study, BA was demonstrated to increase the activity of serum and hepatic antioxidant enzymes in broilers. Dietary BA supplementation contributed to enhancing the antioxidant capacity of broilers, thereby counteracting adverse outcomes induced by oxidative stress during production. Other studies have shown that antioxidant additives can effectively alleviate oxidative stress in broilers [31,32]. However, oxidative stress-challenged models (e.g., diquat, dexamethasone) were not adopted in the current experiment. The therapeutic effects of BA under specific stress conditions remain to be further investigated.

PPARα acts as a key transcriptional regulator of hepatic lipid catabolism; it transcriptionally upregulates the lipolytic gene *HSL* while suppressing lipogenic genes including *ACC* and *FASN* [33,34]. HSL promotes lipid breakdown [35], whereas ACC and FASN are critical rate-limiting enzymes driving de novo fatty acid synthesis [36]. Collectively, this regulatory axis balances lipolysis and lipogenesis to maintain hepatic lipid homeostasis. In the present study, BA effectively increased the protein levels of phosphorylated PPARα and HSL while decreasing FASN in the broiler liver, confirming the regulatory effects of BA on hepatic lipid metabolism. When detecting ACC protein, primary antibodies from multiple commercial suppliers were tested, yet no obvious immunoblot bands were obtained. This could be attributed to the poor antibody specificity against the avian ACC protein. Therefore, ACC protein data were not presented in the Western blot results. In addition, future in vitro studies involving the knockdown or overexpression of key PPARα-related genes are needed to further clarify the lipid-regulating function of BA. Furthermore, the transcriptomic results showed that carbon metabolism, glycine–serine–threonine metabolism, and the phagosome pathway were markedly enriched in the BA group versus the control group, in addition to the PPARα signaling pathway. Further work is required to decipher the mechanisms of BA via these pathways.

In the combined analysis of broiler plasma metabolites and intestinal microbiota sequencing, concurrent elevations in the genera *Caecibacterium* and *Lachnospiraceae* and the metabolite isoleucyl-glutamate attracted our attention. Isoleucyl-glutamate is a dipeptide synthesized from the condensation of the branched-chain amino acid isoleucine and glutamic acid, and it serves as a critical substrate for butyrate synthesis. Intestinal dipeptide abundance directly influences the substrate utilization efficiency of butyrate-producing bacteria [37]. Existing studies have confirmed that key butyrate-producing microbes colonizing the cecum participate in short-chain fatty acid synthesis; metabolites produced by these microbes can cross the intestinal epithelium into systemic circulation, thereby altering serum short-chain fatty acid levels [38,39]. In mouse model research, BA-driven shifts in gut-microbial metabolites, particularly butyric acid, has been shown to alter plasma butyric acid levels and contribute to its systemic effects [40]. Meanwhile, butyric acid is the energy source for cecal epithelial cells. Adequate butyric acid can maintain the height of villi and the normal morphology of crypts and repair intestinal damage [41]. Butyric acid can even activate AMPK-induced mitochondrial phagocytosis through the AMPK–mitophagy pathway, alleviating oxidative stress [42]. Consistent with the above mechanism, the plasma butyric acid concentration was also significantly higher in the BA group than in the control group in the present study, while cecal butyric acid showed no obvious statistical difference. Such an observation raises the possibility that the change in circulating butyric acid may not simply result from greater cecal butyrate synthesis. It could be related to shifts in intestinal absorption, hepatic uptake, or systemic metabolic turnover, rather than merely enhanced cecal butyric acid production. Future studies could directly investigate the effects of butyric acid on broiler health. In addition, it is necessary to verify whether the beneficial effects of BA would be altered when butyric acid production is suppressed, so as to further clarify the underlying mechanism of BA.

There are several limitations to the present study. (1) Only one supplemental dosage of BA was used in the animal trial, and dose gradient groups were not established. Therefore, the optimal supplemental dosage of BA for white-feathered broilers remains to be further explored. (2) Only the jejunum was assessed for intestinal-related phenotypes; hence, the potential effects of BA on the duodenum and ileum remain unclear. (3) Microbiota profiling was conducted at a single time point, so data reflecting dynamic temporal shifts in the gut microbiota are lacking. (4) Although this study explored the role of BA in regulating the hepatic PPARα signaling pathway, the molecular mechanisms underlying the effects of BA on intestinal health were not investigated. (5) For the combined analysis of the gut microbiota and serum metabolites, only Spearman’s correlation analysis was performed. This approach is purely descriptive and cannot establish causal links among gut bacteria, serum metabolites, and biological functions. Whether causal relationships exist between BA-induced shifts in serum metabolites and the gut microbiota remains unknown. Overall, further research is still required to fully elucidate the effects of BA on liver and intestinal health in white-feathered broilers.

## 5. Conclusions

Dietary supplementation with 100 mg/kg BA improved hepatic and intestinal health in white-feathered broilers. BA enhanced the serum and hepatic antioxidant capacity and regulated hepatic lipid metabolism via the PPARα signaling pathway. It also increased the intestinal villus/crypt ratio and digestive enzyme activity, with concomitant gut microbiota remodeling marked by the enrichment of beneficial *Lachnospiraceae* and shifts in butyric acid-related metabolic profiles. Collectively, the findings of this study provide experimental evidence supporting the application of BA for improving hepatic and intestinal health in broiler production.

## Figures and Tables

**Table 1:** Composition and nutrient levels of diets (air-dry basis).

Item	1 to 21 Days of Age	22 to 42 Days of Age
Ingredient/%		
Corn	51.75	53.26
Soybean meal	34.73	31.37
Extruded soybean	3.00	6.20
Fish meal	2.00	0.00
Soybean oil	4.21	5.00
Limestone	1.22	1.15
CaHPO_4_	1.53	1.56
DL-Methionine	0.26	0.16
NaCl	0.30	0.30
Premix ^1^	1.00	1.00
Total	100	100
Nutrient level ^2^		
Metabolic energy/MJ·kg^−1^	12.56	12.97
Crude protein/%	21.5	20
Ca/%	1	0.9
Total *p*/%	0.45	0.4
Lysine/%	1.19	1.08
Methionine/%	0.59	0.45
Methionine + cysteine/%	0.91	0.76
Threonine/%	0.82	0.76

^1^ The premix provided the following per kg of diet: (1 to 21 days of age) vitamin A 9800 IU, vitamin D3 2780 IU, vitamin E 19.6 mg, vitamin K3 2.24 mg, vitamin B1 1.4 mg, vitamin B2 7 mg, vitamin B3 33.6 mg, vitamin B5 9.8 mg, vitamin B12 0.0168 mg, pyridoxine 3.36 mg, biotin 0.056 mg, folic acid 1.12 mg, choline 1300 mg, Cu 8 mg, Fe 100 mg, Mn 120 mg, Zn 100 mg, Se 0.3 mg, I 0.7 mg; (22 to 42 days of age) vitamin A 7000 IU, vitamin D3 2700 IU, vitamin E 14 mg, vitamin K3 1.6 mg, vitamin B1 1 mg, vitamin B2 5 mg, vitamin B3 24 mg, vitamin B5 7 mg, vitamin B12 0.0168 mg, pyridoxine 2.40 mg, biotin 0.04 mg, folic acid 0.8 mg, choline 1000 mg, Cu 8 mg, Fe 80 mg, Mn 100 mg, Zn 80 mg, Se 0.3 mg, I 0.7 mg. ^2^ Metabolic energy is the calculated value, while the others are the measured values. The basal diet was formulated following the Chinese national standard GB/T 5916-2020. Crude protein content was assayed using the Kjeldahl digestion–distillation method. Calcium content was quantified by atomic absorption spectrophotometry. Total phosphorus was determined via molybdenum–vanadate spectrophotometry. Amino acids (lysine, methionine, cysteine, and threonine) were measured by high-performance liquid chromatography (HPLC) after acid hydrolysis. Metabolizable energy was calculated from the tabulated nutritional values of individual feed ingredients.

**Table 2:** The effects of BA on the growth performance of broilers.

Item	Group		*p*-Value
	Control	BA 100 mg/kg	
1 to 21 days of age			
IBW, g	44.23 ± 0.57	43.64 ± 0.19	0.117
FBW, g	856.52 ± 2.68	886.80 ± 41.40	0.067
ADG, g/d	40.61 ± 0.15	42.16 ± 2.08	0.068
ADFI, g/d	54.97 ± 0.30	54.70 ± 0.65	0.153
FCR	1.35 ± 0.004	1.31 ± 0.03	0.134
22 to 42 days of age			
FBW, g	3070.31 ± 32.41	3044.26 ± 82.87	0.252
ADG, g/d	105.42 ± 1.19	102.74 ± 2.92	0.107
ADFI, g/d	140.05 ± 3.80	138.55 ± 2.44	0.443
FCR	1.33 ± 0.05	1.35 ± 0.06	0.668
1 to 42 days of age			
ADG, g/d	71.45 ± 1.38	72.81 ± 1.36	0.925
ADFI, g/d	98.55 ± 1.83	97.02 ± 0.57	0.135
FCR	1.38 ± 0.01	1.33 ± 0.03	0.074

Data are presented as the mean ± standard deviation (SD). IBW, initial body weight; FBW, final body weight; ADG, average daily gain; ADFI, average daily feed intake; FCR, feed conversion ratio; BA, baicalin.

## Data Availability

The original contributions presented in the study are included in the article/Supplementary Materials. Further inquiries can be directed to the corresponding author.

## Supplementary Materials

The following supporting information can be downloaded at https://www.mdpi.com/article/10.3390/biology15181569/s1. Figure S1: The structural formula of baicalin; Table S1: Primer sequences used in this study; File S1: WB original images of Figure 4.

## Abbreviations

The following abbreviations are used in this manuscript:

BA	Baicalin
SOD	Superoxide dismutase
GSH-Px	Glutathione peroxidase
CAT	Catalase
GSH	Glutathione
Nrf2	Nuclear factor erythroid 2-related factor 2
HO-1	Heme oxygenase-1
NF-κB	Nuclear factor-kappa B
IBW	Initial body weight
FBW	Final body weight
ADG	Average daily gain
ADFI	Average daily feed intake
FCR	Feed conversion ratio
TAC	Total antioxidant capacity
MDA	Malondialdehyde
DEGs	Differentially expressed genes
TPM	Transcripts per million
SRA	Sequence Read Archive
RT-qPCR	Real-time quantitative PCR
VH	Villus height
CD	Crypt depth
GSTT1L	Glutathione S-transferase theta 1-like
NQO1	NAD(P)H: quinone oxidoreductase 1
MPO	Myeloperoxidase
FAS	Fatty acid synthase
PPARα	Peroxisome proliferator-activated receptor α
CPT1	Carnitine palmitoyltransferase 1
ACC	Acetyl-CoA carboxylase
HSL	Hormone-sensitive lipase
Glu	Glutamic acid
ROS	Reactive oxygen species
AMPK	Adenosine 5′-monophosphate-activated protein kinase
