# Supplementary material for: Baicalin Modulates Hepatic Lipid Metabolism and Improves Intestinal Health in White-Feathered Broilers
Source: Biology (Basel). 2026 Sep 8;15(18):1569. doi: 10.3390/biology15181569 (PMC13603962; doi:10.3390/biology15181569)
Supplement: Supplementary file 1 [file biology-15-01569-s001.zip › Supplementary Figure S1.pdf]

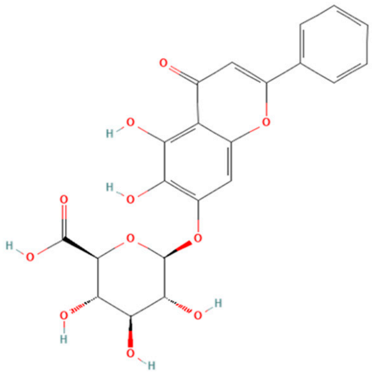

Supplementary Figure S1. The structural formula of baicalin.
